# Machine learning-based prediction model for myocardial ischemia under high altitude exposure: a cohort study

**DOI:** 10.1038/s41598-024-51202-8

**Published:** 2024-01-06

**Authors:** Yu Chen, Xin Zhang, Qing Ye, Xin Zhang, Ning Cao, Shao-Ying Li, Jie Yu, Sheng-Tao Zhao, Juan Zhang, Xin-Ming Xu, Yan-Kun Shi, Li-Xia Yang

**Affiliations:** 1 Department of Cardiology, 920th Hospital of Joint Logistics Support Force, PLA, Kunming, China; 2 Department of Pulmonary and Critical Care Medicine, 920th Hospital of Joint Logistics Support Force, PLA, Kunming, China; 3 Department of Radiology, 920th Hospital of Joint Logistics Support Force, PLA, Kunming, China; 4 Department of Neurosurgery, 920th Hospital of Joint Logistics Support Force, PLA, Kunming, China; 5 Department of Thoracocardiac Surgery, 920th Hospital of Joint Logistics Support Force, PLA, Kunming, China; 6 Department of Quality Control, 920th Hospital of Joint Logistics Support Force, PLA, No. 212 Daguan Rd, Kunming, 650032 Yunnan China; 7 Department of Radiation Oncology, First Affiliated Hospital of Kunming Medical University, Kunming, China

**Keywords:** Computational biology and bioinformatics, Cardiology, Health occupations

## Abstract

High altitude exposure increases the risk of myocardial ischemia (MI) and subsequent cardiovascular death. Machine learning techniques have been used to develop cardiovascular disease prediction models, but no reports exist for high altitude induced myocardial ischemia. Our objective was to establish a machine learning-based MI prediction model and identify key risk factors. Using a prospective cohort study, a predictive model was developed and validated for high-altitude MI. We consolidated the health examination and self-reported electronic questionnaire data (collected between January and June 2022 in 920th Joint Logistic Support Force Hospital of china) of soldiers undergoing high-altitude training, along with the health examination and second self-reported electronic questionnaire data (collected between December 2022 and January 2023) subsequent to their completion on the plateau, into a unified dataset. Participants were subsequently allocated to either the training or test dataset in a 3:1 ratio using random assignment. A predictive model based on clinical features, physical examination, and laboratory results was designed using the training dataset, and the model's performance was evaluated using the area under the receiver operating characteristic curve score (AUC) in the test dataset. Using the training dataset (n = 2141), we developed a myocardial ischemia prediction model with high accuracy (AUC = 0.86) when validated on the test dataset (n = 714). The model was based on five laboratory results: Eosinophils percentage (Eos.Per), Globulin (G), Ca, Glucose (GLU), and Aspartate aminotransferase (AST). Our concise and accurate high-altitude myocardial ischemia incidence prediction model, based on five laboratory results, may be used to identify risks in advance and help individuals and groups prepare before entering high-altitude areas. Further external validation, including female and different age groups, is necessary.

## Introduction

High altitude increases the risk of myocardial ischemia^1^, primarily caused by occlusive coronary artery disease, coronary microvascular dysfunction, or both. Exposure to high altitudes leads to reduced arterial oxygen saturation, worsening pre-existing cardiovascular disease^1^. In patients with coronary artery disease living at high altitudes, myocardial ischemia is driven by increased myocardial oxygen demand due to elevated heart rate, enhanced contractility, and increased afterload. This imbalance can worsen ischemia due to respiratory alkalosis or coronary artery vasoconstriction secondary to hypoxia or spasm^2,3^.

Currently, there is a scarcity of epidemiological data pertaining to populations exposed to high altitudes and the incidence of myocardial ischemia. Soldiers who are frequently obliged to undergo training and perform operations in high altitude areas for mission purposes, are vulnerable to both acute and chronic cardiovascular events. The ability to accurately predict the occurrence of myocardial ischemia in individuals entering high altitude environments would facilitate early interventions or appropriate personnel selection.

In this study, we aim to fill this gap by establishing a machine learning-based predictive model for the occurrence of myocardial ischemia within six months of entering high altitudes and identifying the key clinical features that significantly contribute to the model's predictive capabilities. By providing a reliable predictive model and identifying crucial clinical features, this study will aid in ensuring the safety of military personnel and others who are required to enter high altitude environments.

## Methods

### Study design and population

In this study, we employed a prospective cohort study design. The sample population was drawn from soldiers who received health examinations at the 920th Hospital of the Joint Logistic Support Force between January 2022 and June 2022, and who were scheduled to undergo high-altitude training (at an altitude of 3000–3500 m) within six months. Inclusion criteria: 1. Male and female military personnel aged ≥ 18 & ≤ 60; 2. Underwent health examination at the 920th Hospital between January 2022 and June 2022; 3. Scheduled to undergo high-altitude training within 6 months of health examination; 4. Completed first self-reported questionnaire; 5. Completed second health examination and questionnaire at high-altitude training site. Exclusion criteria: 1. History of structural heart disease, hypertension, coronary artery disease, chronic obstructive pulmonary disease, pneumonia, asthma; 2. Abnormal ECG findings. 3. Did not ultimately participate in high-altitude training abnormal electrocardiogram; 4. Developed acute mountain sickness, severe training injuries, or COVID-19 during study period.

**Figure 1:**
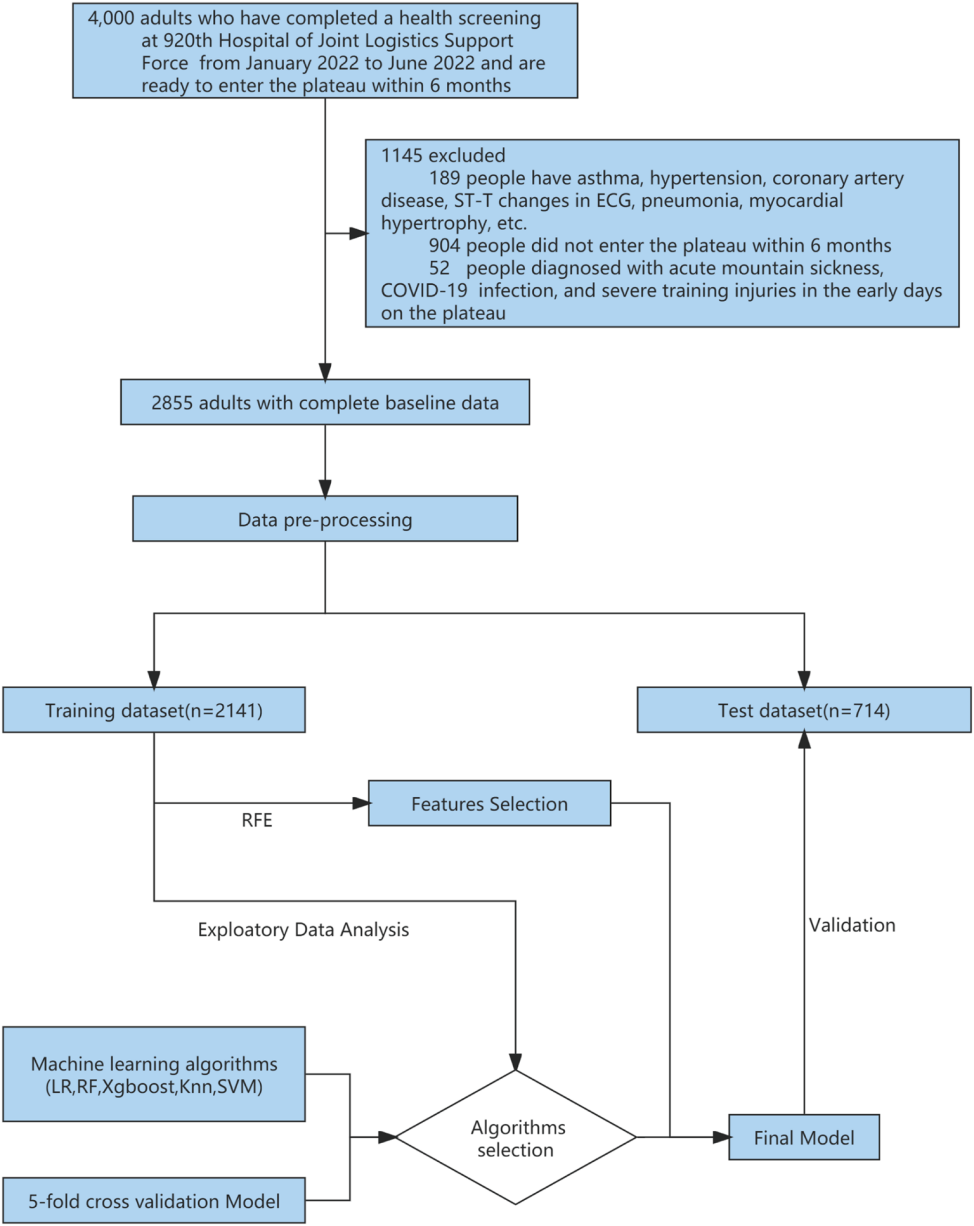
Workflow for data management and myocardial ischemia prediction model development. Data came from 4000 adults who completed physical exams at the 920th Hospital of Joint Logistics Support Force between January and June 2022 and prepared to enter the plateau within 6 months. After exclusion, there were 2855 people remaining. Participants' data (n = 2855) were randomly assigned to a prediction model training dataset (n = 2141) and a test dataset (n = 714) in a 3:1 ratio following preprocessing. fivefold cross-validation was used for training and selecting the prediction model, and five classification algorithms were evaluated. Feature selection was conducted using the RFE (Recursive Feature Elimination) algorithm. The final prediction model was validated using a test dataset. *LR* logistic regression, *RF* random forest, *XGBoost* eXtreme gradient boosting, *KNN* K-nearest neighbor, *SVM* support vector machines.

In total, 4000 individuals participated in the health examination, of whom 3800 were men and 200 were women aged 18 to 54 years old. The examination included chest X-rays, electrocardiograms, ultrasounds, and hematological tests. After careful screening, we excluded a total of 1093 individuals from the study. Specifically, we excluded 189 cases (both male and female) with a history of pneumonia, asthma, hypertension, abnormal electrocardiogram findings (including ST-T changes, abnormal Q waves, and arrhythmia), and myocardial hypertrophy, as well as 904 individuals who did not ultimately participate in high-altitude training. The remaining 2907 soldiers completed the first self-reported electronic questionnaire, which included smoking status, duration and amount of smoking, recent physical ability test (3 km test, Sit-ups and Serpentine Run) scores (Table 1), Chesttightness or Chestpain, altitude of residence, education level, marital status, and content from the Scale for Outcomes in Parkinson′s Disease for Autonomic Symptom (SCOPA-AUT) score^4^. A medical team performed a health examination on the population at the high-altitude training site from December 2022 to January 2023, which included chest X-rays, electrocardiograms, heart rate (HR), systolic blood pressure (SBP) and diastolic blood pressure (DBP), oxygen saturation (OS), and a second self-reported electronic questionnaire. We also obtained electronic medical records from the previous six months and excluded 52 individuals with acute mountain sickness, severe training injuries, or COVID-19 infections, resulting in a final sample of 2855 individuals, including 2810 men and 45 women (Fig. 1; Additional file 1).

**Table 1:** Characteristics of participants in the training and test datasets.

	Training dataset				Test dataset			
	No	Yes	OR	P	No	Yes	OR	P
	*N* = 1964	*N* = 177	(95% CI)		*N* = 656	*N* = 58	(95% CI)	
Age (y)	23.0 [22.0; 26.0]	24.0 [22.0; 29.0]	1.07 [1.04; 1.10]	0.001	24.0 [22.0; 27.0]	24.0 [22.0; 27.8]	1.03 [0.97; 1.09]	0.313
Sex:				0.005				1.000
Female	26 (1.32%)	8 (4.52%)	3.57 [1.48; 7.72]	–	11 (1.68%)	0 (0.00%)		–
Male	1938 (98.7%)	169 (95.5%)	Ref	–	645 (98.3%)	58 (100%)		–
Smoking duration				0.012				0.274
None	1073 (54.6%)	92 (52.0%)	Ref	–	384 (58.5%)	28 (48.3%)	Ref	–
< 10 years	779 (39.7%)	65 (36.7%)	0.97 [0.70; 1.35]	–	218 (33.2%)	25 (43.1%)	1.57 [0.89; 2.77]	–
≥ 10 years	112 (5.70%)	20 (11.3%)	2.09 [1.21; 3.46]	–	54 (8.23%)	5 (8.62%)	1.30 [0.42; 3.27]	–
Chesttightness.or.Chestpain				0.002				0.268
No	1867 (95.1%)	158 (89.3%)	Ref	–	614 (93.6%)	52 (89.7%)	Ref	–
Yes	97 (4.94%)	19 (10.7%)	2.33 [1.35; 3.83]	–	42 (6.40%)	6 (10.3%)	1.72 [0.62; 3.98]	–
BMI (kg/m^2^)	22.1 [20.8; 23.6]	22.6 [21.0; 24.4]	1.09 [1.02; 1.16]	0.023	22.2 [20.8; 23.8]	22.5 [20.7; 23.7]	1.02 [0.90; 1.15]	0.680
SBP (mmHg)	116 [110; 120]	118 [110; 124]	1.00 [1.00; 1.01]	0.018	118 [110; 121]	120 [110; 130]	1.04 [1.02; 1.07]	0.006
DBP (mmHg)	80.0 [73.0; 84.0]	80.0 [75.0; 85.0]	1.01 [0.99; 1.03]	0.436	80.0 [73.0; 84.0]	81.5 [77.0; 88.0]	1.05 [1.02; 1.08]	0.002
HR (min^−1^)	80.0 [71.0; 89.0]	80.0 [75.0; 88.0]	1.00 [0.99; 1.02]	0.537	79.0 [70.0; 88.0]	78.5 [69.0; 85.8]	0.99 [0.97; 1.01]	0.486
OS (%)	93.0 [92.0; 95.0]	93.0 [92.0; 96.0]	1.01 [0.97; 1.04]	0.159	93.0 [92.0; 95.0]	93.0 [92.0; 95.0]	1.00 [0.99; 1.01]	0.132
Highland acclimatization training*				0.986				1.000
No	151 (7.69%)	13 (7.34%)	Ref	–	37 (5.64%)	3 (5.17%)	Ref	–
Yes	1813 (92.3%)	164 (92.7%)	1.04 [0.60; 1.97]	–	619 (94.4%)	55 (94.8%)	1.05 [0.36; 4.61]	–
Altitude of original station (m)				0.002				0.202
< 1500	1830 (93.2%)	152 (85.9%)	Ref	–	593 (90.4%)	50 (86.2%)	Ref	–
1500 –2500	114 (5.80%)	23 (13.0%)	2.44 [1.48; 3.87]	–	51 (7.77%)	8 (13.8%)		–
> 2500	20 (1.02%)	2 (1.13%)	1.29 [0.19; 4.49]	–	12 (1.83%)	0 (0.00%)		–
3 km test in Non-High Altitude (Score)^#^	77.0 [65.0; 91.0]	82.0 [62.0; 93.0]	1.00 [0.99; 1.00]	0.414	77.0 [65.0; 91.0]	81.0 [0.00; 90.0]	1.00 [0.99; 1.00]	0.944
Sit-ups in Non-High Altitude (Score)^#^	93.0 [85.0; 101]	95.0 [88.0; 101]	1.01 [1.00; 1.02]	0.173	93.0 [85.0; 101]	93.0 [86.2; 101]	1.00 [0.98; 1.02]	0.814
Serpentine Run in Non-High Altitude (Score)^#^	90.0 [75.0; 96.0]	90.0 [78.0; 96.0]	1.00 [0.99; 1.01]	0.923	90.0 [75.0; 96.0]	83.0 [72.5; 95.0]	0.99 [0.98; 1.00]	0.195
SCOPA-AUT SCORE in non-high altitude	4.00 [2.00; 8.00]	3.00 [2.00; 7.00]	0.98 [0.95; 1.02]	0.170	4.00 [2.00; 8.00]	5.00 [3.00; 8.00]	1.03 [0.97; 1.10]	0.121
Ca (mmol/L)	2.37 [2.31; 2.41]	2.42 [2.32; 2.47]	2.23 [1.88; 3.63]	< 0.001	2.37 [2.30; 2.41]	2.38 [2.31; 2.47]	1.75 [1.04; 2.32]	0.036
Crea (μmol/L)	88.0 [80.0; 97.0]	83.0 [79.0; 93.0]	0.97 [0.95; 0.98]	< 0.001	89.0 [81.0; 97.0]	85.5 [79.0; 95.0]	0.98 [0.95; 1.01]	0.100
GLU (mmol/L)	4.03 [3.53; 4.47]	4.33 [4.10; 4.59]	2.11 [1.73; 2.58]	< 0.001	< 0.001	4.03 [3.53; 4.47]	4.33 [3.99; 4.59]	1.92 [1.37; 2.67]
Eos.Per (%)	2.20 [1.60; 3.00]	2.60 [1.30; 5.00]	1.28 [1.20; 1.36]	< 0.001	2.20 [1.50; 3.00]	2.00 [1.10; 4.92]	1.23 [1.10; 1.36]	0.608
G (g/L)	25.6 [23.6; 27.3]	27.8 [25.6; 28.5]	1.22 [1.16; 1.29]	< 0.001	25.6 [22.9; 27.3]	27.8 [25.9; 28.5]	1.20 [1.11; 1.30]	< 0.001
MCHC (g/L)	338 (9.00)	332 (24.5)	0.95 [0.94; 0.97]	0.001	338 (9.25)	335 (10.1)	0.97 [0.94; 1.00]	0.034
P (mmol/L)	1.19 [1.08; 1.27]	1.09 [1.05; 1.14]	0.11 [0.04; 0.31]	< 0.001	1.17 [1.07; 1.25]	1.10 [1.06; 1.14]	0.08 [0.01; 0.47]	0.001
PLT (× 10^9^/L)	230 (37.5)	214 (45.3)	0.99 [0.99; 0.99]	< 0.001	230 (37.7)	211 (49.6)	0.99 [0.98; 0.99]	0.005
AST (U/L)	22.0 [19.0; 30.0]	20.0 [18.0; 26.0]	0.98 [0.96; 0.99]	< 0.001	22.0 [19.0; 29.0]	19.0 [17.0; 24.0]	0.95 [0.92; 0.99]	< 0.001
TP (g/L)	72.0 [70.7; 74.6]	73.7 [70.7; 75.5]	1.11 [1.06; 1.16]	< 0.001	71.5 [69.9; 74.6]	74.1 [70.9; 75.6]	1.11 [1.03; 1.19]	0.014
Urea (mmol/L)	5.10 [4.40; 5.70]	5.50 [5.00; 6.30]	1.35 [1.18; 1.53]	< 0.001	5.10 [4.60; 5.60]	5.90 [5.03; 7.22]	1.65 [1.32; 2.06]	< 0.001
WBC (× 10^9^/L)	6.75 [5.84; 7.36]	5.91 [5.29; 7.26]	0.74 [0.65; 0.85]	< 0.001	6.59 [5.70; 7.36]	5.90 [5.51; 6.86]	0.90 [0.72; 1.13]	0.022

Continuous data conforming to a normal distribution were reported as mean (SD) and those not conforming as median (quartiles). Odds ratios and 95% CIs were calculated, either using count data for categorical variables or a logistic regression model for continuous variables. Statistical significance was determined using t-test, Rank-sum test or χ^2^ test, with p-values calculated.

Non-High Altitude: an altitude < 2500 m. High Altitude: an altitude ≥ 2500 m. *BMI* body mass index, *SBP* systolic blood pressure, *DBP* diastolic blood pressure, *HR* heart rate, *OS* oxygen saturation, *SCOPA-AUT* the Scale for Outcomes in Parkinson′s Disease for Autonomic Symptoms, *Ca* calcium, *Crea* creatinine, *Eos.Per* eosinophils percentage, *G* globulin, *GLU* glucose, *MCHC* mean corpuscular hemoglobin concentration, *P* phosphorus, *PLT* platelet, *AST* aspartate aminotransferase, *TP* total protein, *UA* uric acid, *WBC* white blood cell.

*Participants entered the destination after 1 month of highland acclimatization training at an altitude of 3000 m.

^#^The calculation of physical ability scores was based on Military Common Subject Training Program.

### Outcome variable

In the present study, the diagnosis of myocardial ischemia was determined through evaluation of electrocardiogram (ECG) results, specifically based on the following criteria: (1) the presence of horizontal or downsloping ST depression of greater than 0.5 mm at the J-point in at least two consecutive leads, and (2) the presence of T wave inversion with a depth of at least 1 mm in at least two continuous leads with a dominant R wave (R/S ratio > 1)^5^.

Considering the challenges of large-scale population screening at high altitudes, we are restricted to the use of ECG alone for detecting myocardial ischemia. The definition of myocardial ischemia in this research is based on ECG examinations conducted during resting conditions throughout the high-altitude training period. Participants were required to abstain from any form of physical training, including various sports activities, for a minimum of 48 h prior to the ECG examination.

### Candidate predictors

In this prospective cohort study, we incorporated clinical results from two health examinations conducted prior to and following entry into high-altitude areas as initial variables. Variables such as the number of health examinations and home address were excluded, as were highly correlated variables such as education level and marital status. Univariate analysis was performed and only hematological test results with a P-value < 0.001 were retained in the final variables. Subsequently, feature selection and engineering were applied to select the most pertinent features and create new variables that could prove useful for our predictive model. To achieve our goal of predicting the risk of myocardial ischemia upon entering high-altitude areas, we included 27 variables in our machine learning analysis, such as age, gender, body mass index (BMI), SBP, DBP, HR, OS, Highland acclimatization training, Altitude of original station, 3 km test in Non-High Altitude (Score), Sit-ups in Non-High Altitude (Score), Serpentine Run in Non-High Altitude (Score), and SCOPA-AUT SCORE in Non-High Altitude (Table 1).

### Sample size estimation

The use of machine learning models in predicting binary clinical outcomes has gained significant attention in recent years, but determining the appropriate sample size for these models remains a challenge. While no established method for determining sample size currently exists, a conventional approach suggests a minimum of 10 outcome events per variable for building a binary clinical outcome prediction model^6^. In this study, we utilized the R pmsampsize package to calculate a sample size of 1100 for a traditional logistic regression model and our study utilized a sample size greater than this calculation.

### Datasets

We preprocessed the data by standardizing it and dividing it randomly into a training set (75%) and a test set (25%). The outcome variable was dichotomized, and continuous and categorical variables were analyzed using appropriate statistical tests. Significant variables (p < 0.001) were included in subsequent analysis focused on hematological findings. Prediction models were constructed using algorithms and features and evaluated in the test set. The analysis was conducted using R software.

### Identification and validation of the prediction model

Our study aims to develop a machine learning model to categorize individuals as having "myocardial ischemia" or not. Model efficacy will be evaluated using the area under the receiver operating characteristic curve (AUC)^7^. The dataset will be randomly divided into training and test sets using a stratified sampling approach. We will use LR^8^, RF^9^, XGBoost^10^, KNN^11^, and SVM^12^ algorithms to fit models to the training set and validate them on the test set. The best algorithm will be selected based on AUC scores and calibration curve performance.

We used the Recursive Feature Elimination (RFE) algorithm^13^ to identify significant variables for a more optimal and clinically feasible model. The resulting model, based on the most influential variables, was compared to the full dataset model and found to be well-suited for practical use.

We used the tidymodels 1.0.0 framework and R programming language (version 4.2.0), along with tidyverse, tidymodels, and caret packages for data analysis, implementing fivefold cross validation (Additional file 1).

### Ethics approval and consent to participate

In accordance with the ethical guidelines of the Helsinki Declaration, the experimental protocol was developed and approved by the Human Ethics Committee of the 920th Hospital of the Joint Logistics Support Force. (Lot no. 2022-135-01). Written informed consent was obtained from individual or guardian participants.

## Results

In our study, univariate analysis was used to identify clinical and hematological factors associated with myocardial ischemia. Results showed that individuals with myocardial ischemia were older, had a higher BMI and SBP, and tended to be female (OR 3.57, 95% CI 1.48–7.72, p = 0.005) and experience chest tightness or pain (OR 2.33, 95% CI 1.35–3.83, p = 0.002). Smoking duration for more than 10 years (OR 2.33, 95% CI 1.35–3.83, p = 0.002) was also found to be a risk factor for myocardial ischemia. Furthermore, individuals living at 1500–2500 m (OR 2.44, 95% CI 1.48–3.87, p = 0.002) experienced a greater risk of myocardial ischemia than those at 1500 m. Interestingly, scores of physical ability test and SCOPA-AUT did not differ significantly between the two groups. Among hematological markers, we identified 12 indicators that showed significant differences (p < 0.001) between the two groups, including Ca, Crea, Eos.Per, G, GLU, MCHC, P, PLT, AST, TP, Urea, and WBC. These factors were used to develop our algorithm model, though other 11 markers such as ALB, AST, ALP, and Eo also showed differences between the two groups (P < 0.05) (Additional file 2: Table S1).

**Figure 2:**
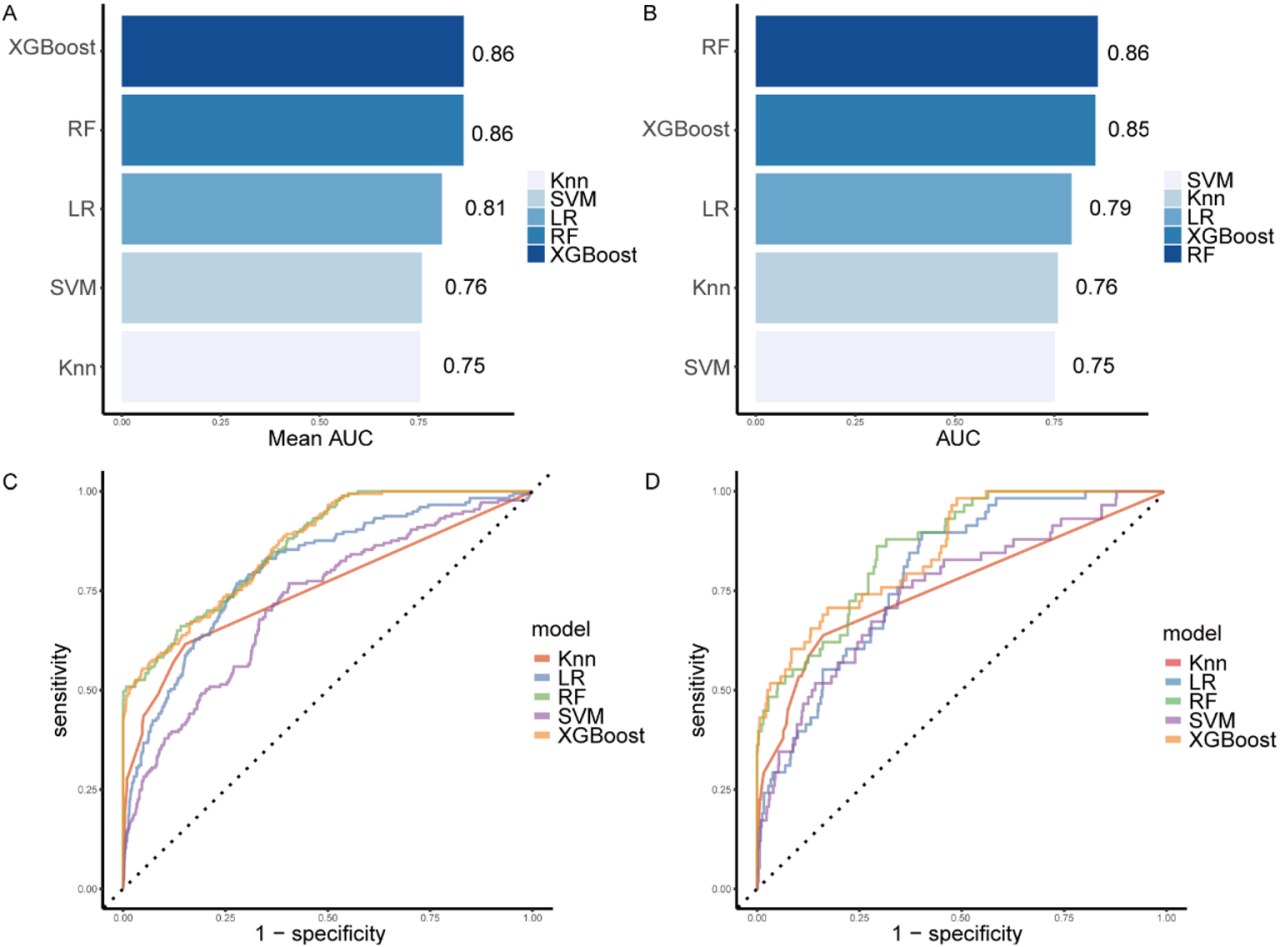
Results from the model training and algorithm selection process. (**A**) The bar chart shows the average AUC score for each candidate algorithm in the training set. (**B**) The bar chart shows the AUC score of the best model for each candidate algorithm in the test set. (**C**) The best ROC curve for each candidate algorithm in the training set. (**D**) ROC plot of the best model for each candidate algorithm in the test set. Details of these analysis methods are provided in the appendix (Additional file 2: Table S2). *AUC* area under the receiver operating characteristic curve, *ROC* Receiver operating characteristic (ROC) curve.

All analyzed models exhibited high performance in predicting the outcome, with RF and XGBoost algorithms models outperforming the other three models. Both the mean AUC for models of RF in the training dataset and AUC for the model of RF in the test dataset were 0.86, demonstrating superior discrimination ability of RF. The accuracy and precision scores for all models were also provided for comprehensive evaluation (Additional file 2: Table S2). Overall, the RF model demonstrated the best predictive performance in both the training and test datasets (Fig. 2).

**Figure 3:**
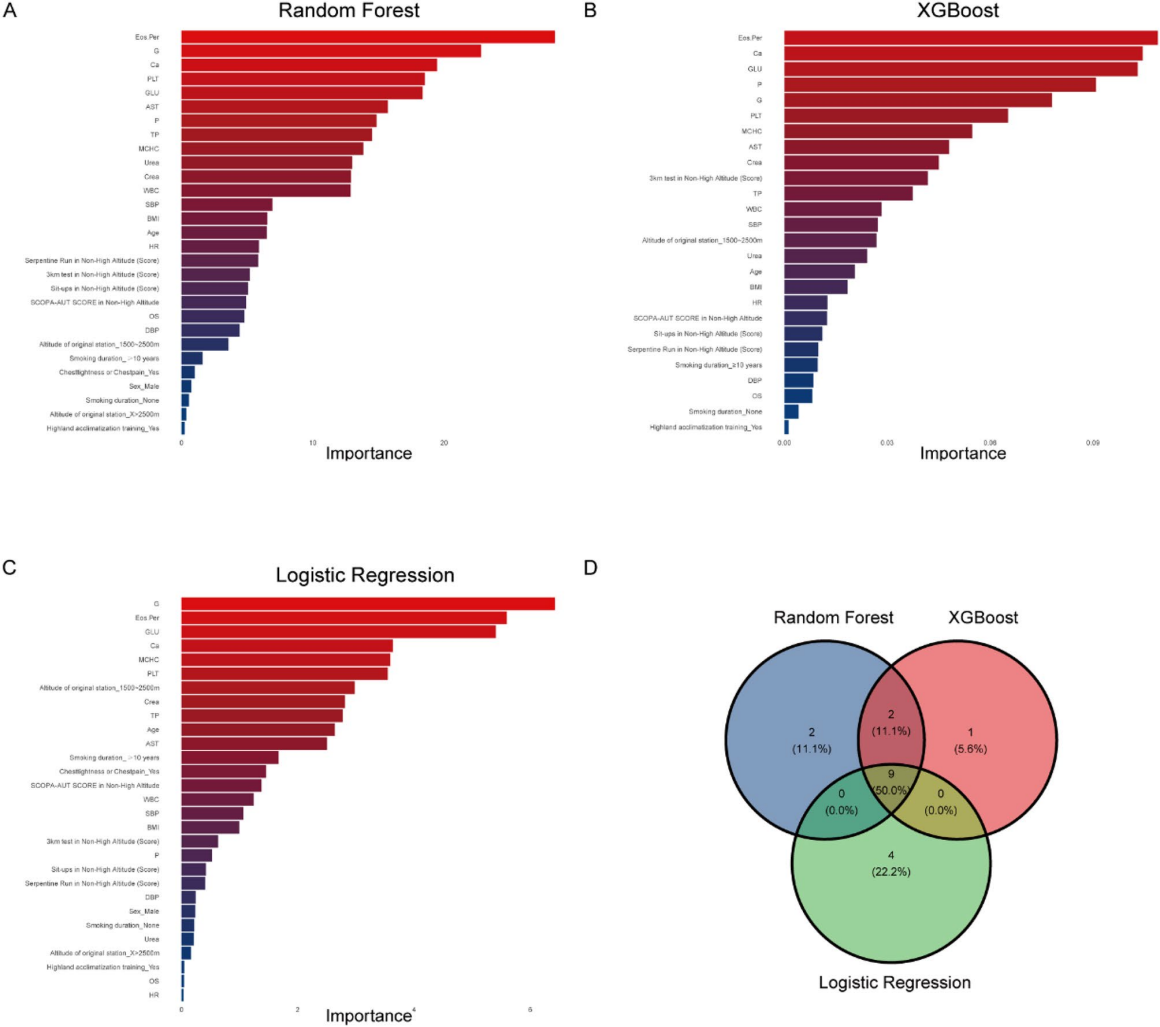
Variable importance scores of RF, XGBoost and LR. (**A**)–(**C**) Variable importance scores of RF, XGBoost and LR, respectively. Details of these analysis methods are provided in the appendix (Additional file 3: Table S3). (**D**) The Venn diagram of the top 50% ranked variables in the RF, XGBoost and LR algorithm models, with 9 intersecting variables including Eos.Per, G, Ca, PLT, GLU, AST, TP, MCHC, and Crea.

In developing a machine learning model, many variables are considered, but not all variables are equally important. To determine the important variables and evaluate risk factors that influence the outcome, variable importance plots were utilized to rank the variables according to their importance, and we found that 27 important clinical features were able to predict myocardial ischemia in the RF and LR algorithm models, while the XGboost algorithm model identified 25 important clinical features. After analyzing the top 50% of clinical features from three algorithm models, we found that 9 variables were consistently identified as the most important. These variables, including Eos.Per, Ca, GLU, G, PLT, MCHC, AST, Crea, and TP were deemed critical for the predictive model (Fig. 3).

**Figure 4:**
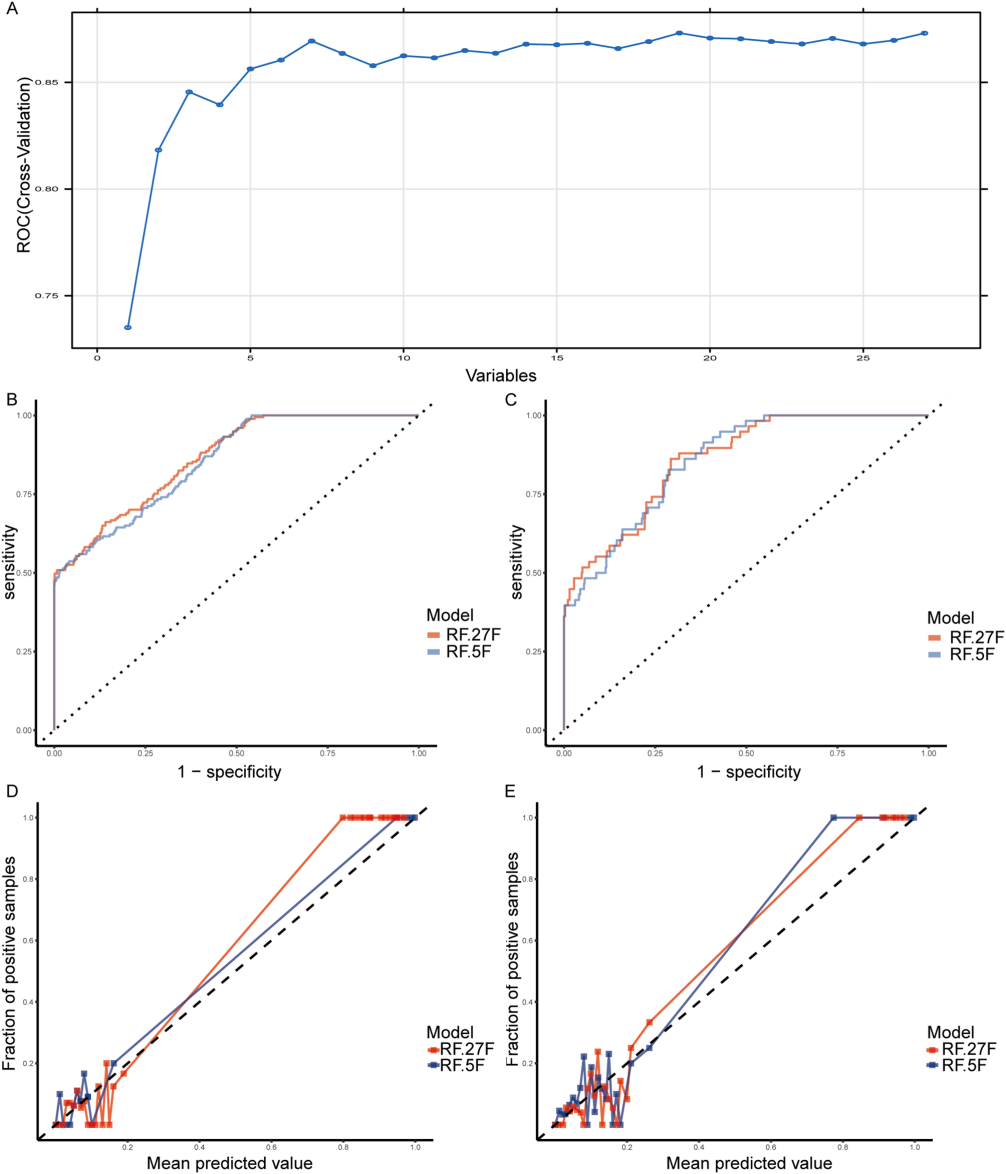
AUC versus the number of variables used in RFE and performance comparison of 27F model and 5F model under RF algorithm. (**A**) The performance of RFE with respect to different variables using the area under the ROC curves. The graph illustrates that the model achieved comparable performance with the highest AUC by utilizing 5 selected features. (**B**) The ROC curves of the 27F and 5F models based on the training dataset. On (**C**), we have shown the ROC curves for the 27F and 5F models for the testing dataset. The calibration curves for the 5F and 27F models for the training dataset (**D**) and test dataset (**E**) are presented.

To enhance the clinical feasibility of the RF algorithm in predicting myocardial ischemia, we investigated whether a smaller subset of 27 features could yield a more precise model. By utilizing the RFE method, we evaluated the performance of the RF algorithm with varying numbers of features selected from the full set of 27. Intriguingly, our findings indicated that the RF algorithm demonstrated a turning point in AUC with as few as 5 features (Fig. 4). Notably, the 5 features identified from the training dataset were Eos.Per, G, Ca, GLU, and AST. These results suggest that a simplified model containing only a small number of crucial features may be useful in a clinical setting.

Building on our findings, we developed a second prediction model using the RF algorithm in the training set, which refer to as the 5F model. We then compared its performance with the previously constructed 27F model. Both models performed well in the training dataset (mean AUC scores of 0.86 and 0.85, respectively) and showed similar performance in the test set (AUC score of 0.86). The calibration curves of the 5F and 27F models on both datasets demonstrated good predictive ability for myocardial ischemia (Fig. 4). In the training dataset, the calibration slopes of the 5F model (1.31, 95% CI 0.38–2.24) and 27F model (0.90, 95% CI 0.68–1.12) were compared. However, in the test set, the calibration slopes of the two models were both favorable (1.17, 95 CI 0.81–1.54 vs 0.84, 95% CI 0.62–1.05). Notably, the slopes of all curves were relatively close to one, and the intercepts in both datasets were close to zero, suggesting that the established models can feasibly predict myocardial ischemia at high altitude (Additional file 2: Table S5).

Finally, the violin diagram is used to show the comparison of important variable parameters in the 5F model in the overall datasets (Additional file 3: Fig. S1). Prior to entering high altitude, levels of Eos.Per, G, Ca, and GLU were significantly higher in the myocardial ischemia group (p < 0.001), while AST levels were lower in the myocardial ischemia group (p < 0.001). These differences between the two groups were also evident in the testing dataset, with the 5 variables showing distinct performance in predicting myocardial ischemia.

## Discussion

We have successfully developed and validated a predictive model for myocardial ischemia, utilizing clinical and hematological data from 2855 soldiers who underwent training in high-altitude regions (at an altitude of 3000–3500 m), in combination with advanced machine learning algorithms. Our model demonstrated exceptional accuracy (AUC score of 0.86) in both the training and testing sets. While the main risk factors for cardiovascular disease include advanced age, hypertension, type 2 diabetes, hypercholesterolemia, obesity, and smoking^14^, the specific impact and epidemiological situation of these risk factors on cardiovascular disease in high-altitude regions, particularly those above 3000 m, remains largely unknown. Ischemic cardiomyopathy is the primary cause of death in cardiovascular disease, with myocardial ischemia and myocardial infarction being its primary manifestations^15^. A variety of factors, such as emotions, drugs, exercise, and cold exposure, can induce myocardial ischemia events and significantly increase the risk of cardiovascular death or non-fatal myocardial infarction^16–19^. Therefore, the early identification and intervention of the myocardial ischemia process in ischemic cardiomyopathy is of paramount importance^20^. Our high-altitude myocardial ischemia prediction model, based on the machine learning RF algorithm, demonstrates great predictive value for individuals entering high-altitude regions, particularly those above 3000 m. Validation of this model in women, the elderly, and individuals with a history of coronary heart disease could potentially aid even more people.

It is estimated that over 100 million individuals worldwide annually migrate from low-altitude areas to high-altitude regions, with the majority being middle-aged and young adults^21^. The participants in our study were primarily healthy individuals aged 18–54, representing the physically sound middle-aged and young adults who intend to venture into high-altitude regions from the plains. After arriving in these regions, they take on more physically demanding tasks related to basic infrastructure construction, military missions, or high-intensity sports, such as skiing, ice skating, and mountaineering^22^. Our machine learning-based high-altitude myocardial ischemia model can provide more precise predictions for this population. Machine learning techniques have gained widespread use in cardiovascular disease research^23–25^ and are believed to have significant advantages in clinical diagnostic model construction. In our study, we constructed a 5F model (5 main variables: Eos.Per, G, Ca, GLU, AST), which showed no significant AUC difference compared to models with more variables. Fewer clinical features may be more convenient for clinical services.

After healthy individuals ascend to high altitude areas, the cardiovascular system undergoes physiological changes due to the compensatory mechanisms of the body. These changes include an increase in pulmonary artery pressure, blood pressure, heart rate, and changes in cardiac output^26–28^. Coronary heart disease patients also experience corresponding physiological compensatory changes, which may increase the risk of death due to reduced coronary reserve capacity and inadequate oxygen supply. However, the high-altitude environment does not increase mortality rates^29^, indicating that the protective compensatory mechanisms of the heart are effectively resolving these risks. Myocardial cells are protected by these mechanisms by stimulating the sympathetic nervous system, reducing intracellular oxygen pressure, and initiating gene programs throughout the body. These gene programs include antioxidation, anti-inflammatory, anti-apoptosis, and Ca2 + transport proteins^30^. However, the high-altitude environment can intensify numerous cardiovascular disease risk factors, including higher triglycerides, higher cholesterol levels, and lower levels of high-density lipoprotein cholesterol (HDL-C)^31–33^. Blood lipids also change when healthy individuals enter high altitude areas^34^. Additionally, the high-altitude environment can increase systemic inflammatory response factor levels and affect platelet aggregation and fibrinogen levels^35,36^. Nevertheless, activities at high altitude can improve insulin function and maintain blood sugar stability^37^. Modifiable cardiovascular disease risk factors in the high-altitude environment may contribute to the development of high altitude-induced myocardial ischemia. Our research has found that five variables, including Eos.Per, Ca, AST, G, and GLU, may be involved in this process. Eosinophils are important immune cells involved in anti-parasitic and hypersensitivity reactions. It has been suggested that they may promote coronary artery spasm by releasing inflammatory mediators^38^. Clinical studies have also demonstrated that the percentage of eosinophils in peripheral blood is closely related to the subtype and severity of coronary artery disease^39^. Calcium, which is essential for cellular function in the human body, is primarily regulated through renal reabsorption and excretion, intestinal absorption, and bone exchange^40^. The presence of calcium may induce vasoconstriction by affecting nitric oxide and endothelial function, while magnesium, a natural calcium antagonist, can reverse this process^41^. Serum levels of calcium and magnesium have been found to be closely related to coronary artery disease, with the potential to predict the risk of coronary heart disease^42^. AST, a liver transaminase and myocardial biomarker, has unexplored predictive value for myocardial ischemia under plateau exposure^43^. Despite being independently associated with atherosclerosis and cardiovascular events, our study identifies lower AST prior to plateau entry as a risk factor for developing myocardial ischemia on plateau, challenging conventional understanding of AST's role in cardiovascular health. Components of the adaptive immune system, such as lymphocytes and immunoglobulins, are also involved in the development of coronary atherosclerosis^44^. Serum γ-globulin, primarily composed of immunoglobulins, has been found to have prognostic value for stable coronary heart disease patients^45^. Diabetes is a significant risk factor for cardiovascular disease, with changes in blood glucose closely linked to increased risk. Even changes in blood glucose within the normal range are significantly associated with metabolic syndrome, with elderly individuals displaying high normal blood sugar levels at an increased risk of developing cardiovascular disease^46^. Given the evidence, it is speculated that “5F” may enhance the risk factors of high-altitude cardiovascular disease, and that “5F” levels will increase significantly after entering high altitude^37,47^. Further research is necessary to investigate the potential mechanisms underlying the relationship between “5F” changes in high altitude and myocardial ischemia.

This study has certain limitations. Firstly, the majority of our study population were aged 18–35 years old, which may limit the generalizability of our findings to the wider population. Additionally, this age group tends to have a lower incidence of underlying diseases, which may affect the significance of high-altitude myocardial ischemia. Secondly, the male-to-female ratio in our study population was imbalanced, which may limit the representativeness of our results for women. Thirdly, this study location was chosen based on several factors, including the rarity of reports on altitudes above 3000 m, and the challenges associated with conducting health examinations at higher altitudes. While this altitude range may be representative to a certain extent, it is important to note that the number of residents living above 3500 m in China is relatively small.

Fourth, the use of resting ECG as the exclusive diagnostic tool for myocardial ischemia presents inherent limitations, which can escalate the risk of misclassification bias. Despite these limitations, our findings shed light on the clinical and hematological characteristics of high-altitude myocardial ischemia and contribute to a growing body of literature on this important topic.

## Conclusion

With the help of machine learning techniques applied to cohort data of healthy individuals preparing to enter high-altitude regions, we have developed a precise and uncomplicated predictive model for myocardial ischemia. After external validation in diverse populations, including more female participants, the model can aid individuals in identifying their risk and preparing early.

### Supplementary Information

Supplementary Information 1.

Supplementary Tables.

Supplementary Figure S1.

## Data Availability

The datasets generated and/or analysed during the current study are not publicly available due military secrecy. But are available from the corresponding author (email: kzxxm@126.com) on reasonable request.

## Abbreviations

AUC: Area under the receiver operating characteristic curve
Ca: Calcium
Crea: Creatinine
DBP: Diolic blood pressure
Eos.Per: Eosinophils percentage
G: Globulin
GLU: Glucose
HR: Heart rate
KNN: K-nearest neighbor
LR: Logistic regression
MCHC: Mean corpuscular hemoglobin concentration
MI: Myocardial ischemia
OS: Oxygen saturation
P: Phosphorus
PLT: Platelet
RF: Random forest
ROC: Receiver operating characteristic curve
RFE: Recursive Feature Elimination
SBP: Systolic blood pressure
SCOPA-AUT: The Scale for Outcomes in Parkinson′s Disease for Autonomic Symptoms
SVM: Support vector machines
AST: Aspartate aminotransferase
TP: Total protein
UA: Uric acid
WBC: White blood cell
XGBoost: EXtreme Gradient Boosting
