# Supplementary material for: Machine learning-based prediction model for myocardial ischemia under high altitude exposure: a cohort study
Source: Sci Rep. 2024 Jan 6;14:686. doi: 10.1038/s41598-024-51202-8 (PMC10770400; doi:10.1038/s41598-024-51202-8)
Supplement: Supplementary file 1 — Supplementary Information 1. [file 41598_2024_51202_MOESM1_ESM.docx]

Additional description of the process

Each enlisted soldier participating in the physical examination is assigned a Health Examination ID, which serves as a unique identifier for all aspects of the examination. The primary objective of assigning these IDs is to maintain accuracy of the recorded data throughout the duration of the study. Once stationed at the high-altitude plateau, medical records are consistently linked to the corresponding Health Examination ID. To ensure that data is complete and accurate, a thorough review of each electronic questionnaire is conducted by the back-end personnel, with telephone confirmation sought for any missing, duplicated or aberrant data points. At the conclusion of the study, all electronic medical records generated during the study period at 920th Hospital of Joint Logistics Support Force of People's Liberation Army of China, in addition to the medical records related to the second examination and treatment at the high-altitude plateau, are retrieved to enable effective consolidation and analysis of the data.

ECG examination conditions

1. The examination room is maintained at a temperature between 18-25℃ and the examination is conducted on a wooden examination bed.
2. The equipment is operated by two nursing staff who have received extensive training and have over two years of experience in the electrocardiogram examination room.
3. The electrocardiogram machine (EDAN SE-1201,China) was used with standard voltage set at 10 mm/mV and paper speed at 25 mm/s.

(4) Prior to the examination, participants are advised to avoid any strenuous activities and to rest quietly on the examination bed for several minutes. Smokers are requested to abstain from smoking for at least 30 minutes prior to the examination.

Blood pressure measurement conditions

(1) Participants are instructed to rest quietly for at least 5-10 minutes before measuring seated upper arm blood pressure with the arm at heart level.

(2) Blood pressure measurements are performed by trained nurses using a validated upper arm electronic blood pressure monitor (Yuwell YE610A China).

(3) Blood pressure is measured on both arms simultaneously, and the arm with the higher reading is selected for measurement.

(4) Blood pressure measurements are taken twice with a 1-2 minute interval between readings, and the average of the two readings is recorded. If the difference between the systolic or diastolic blood pressure readings is greater than 5 mmHg, the measurements are repeated and the average of three readings is recorded.

Data pre-processing

The raw clinical data from the development set underwent a series of preprocessing steps to ensure appropriate analysis.

1. The inclusion and exclusion criteria, as specified in Figure 1, were applied to the data.

2. Categorical variables were transformed into numerical form based on the order of the discrete values present in the data.

3. Continuous variables were normalized through conversion into their respective z-scores using the formula$：\frac{x-\mu}{\sigma}$: , χ, μ and σ represent the value of a given feature, the mean, and the standard deviation, respectively.

4. Numerical variables with zero variance were removed

5. Predictive variables that showed a correlation greater than 0.7 with other predictive variables were also removed.

The resulting training and test sets were employed in the machine learning analysis described in the following section.

Algorithm selection

In order to determine the optimal machine learning algorithm, the following classification algorithms were used to develop candidate prediction models.

a.Logistic Regression (LR) ^1^is a widely used statistical technique for binary classification problems, where the goal is to predict one of two outcomes based on a set of independent variables. It models the relationship between the dependent variable (the response variable) and a set of independent variables (predictors) using a logistic function. The logistic function transforms the linear relationship between the independent variables and the dependent variable into a probability between 0 and 1. The probability is then used to make predictions about the dependent variable, with a threshold applied to assign an observation to one of two categories.

b. Random Forest^2^ is a machine learning algorithm that is used for both classification and regression problems. It is an ensemble learning method, which means that it combines multiple individual decision trees to form a single predictive model. The algorithm works by constructing multiple decision trees using a random subset of the training data, and then aggregating the predictions from all the individual trees to make the final prediction.

c.XGBoost^3^, which stands for Extreme Gradient Boosting, is an open-source software library for machine learning. It is a gradient boosting algorithm that uses decision trees as the base model for prediction. XGBoost is specifically designed for large-scale, efficient and scalable implementation of gradient boosting, which is why it is widely used in both academia and industry.

d. K-nearest neighbor (KNN)^4^ is a popular machine learning algorithm for classification and regression problems. It operates by identifying the K nearest neighbors of a given sample based on their proximity in the feature space. The algorithm then assigns the most common class among these K neighbors to the sample.

e. Support Vector Machines (SVM)^5^ is a supervised machine learning algorithm that can be used for both classification and regression tasks. SVM is based on the concept of finding the hyperplane that best separates data points into two classes. The hyperplane is determined by maximizing the margin, which is the distance between the closest data points from each class and the hyperplane. These closest data points are known as support vectors, hence the name Support Vector Machines.

In the training set modeling process, the " vfold_cv“ function of the “tidymodels” package was employed to conduct 5-fold cross-validation. This approach involved dividing the training dataset into five non-overlapping subsets, with each subset serving as the validation set in turn. The stratification of the function ensured that the distribution of the target variables was preserved in each fold, leading to a more robust evaluation of the model's performance.

The implementation of the Logistic Regression, KNN, and SVM algorithms was obtained from the tidymodels version 1.0.0, while the implementation of XGBoost was obtained from xgboost version 1.6.0.1, and the implementation of Random Forest was obtained from the ranger package version 0.14.1.

The evaluation of all predictive models was performed using the Area Under the Receiver Operating Characteristic (AUC) score^6^, a widely used metric for evaluating models under the Receiver Operating Characteristic (ROC) curve.

Variable importance

To evaluate the significance of the features in the models, the VIP Ver0.3.2 package was utilized to examine the feature importance of the Logistic Regression (LR), Random Forest (RF), and XGBoost models. The VIP algorithm^7^ is a widely-used feature selection method in predictive modeling, which determines the relative significance of the input features in predicting a response variable. This method is based on the principle of projecting the features onto the response space, and evaluating the importance of each feature based on the variation it contributes to the response space. However, the VIP Ver0.3.2 package does not yet provide support for determining the feature importance of the Support Vector Machine (SVM) and K-Nearest Neighbors (KNN) models.

Features selection

In the context of predictive modeling using clinical data, multiple features may not have relevance to the target problem, and a significant number of features may be redundant^8^. This can result in a reduction of accuracy in predictions, as well as limit the applicability of the model in clinical settings. To address this challenge, we employed the Recursive Feature Elimination (RFE) algorithm to select variables and develop a parsimonious predictive model.

RFE is a widely used feature selection technique in the field of machine learning and data analysis^9^. The technique involves removing the least important features from the dataset and repeating the process until a desired number of features remains. This approach can help to mitigate overfitting and reduce the complexity of the model for improved interpretability and computational efficiency.

For the current study, we utilized the same training set as described in a previous section and the Random Forest algorithm, which was determined to be the optimal classifier. The RFE algorithm was employed to select variables and iteratively refit the model using the remaining predictors. At each iteration, the predictor importances were recalculated, and the predictor with the lowest contribution was eliminated. This process was repeated until a specified number of predictors remained. The evaluation metric used was the Area Under the Receiver Operating Characteristic (AUC) score.

Final prediction model development and validation

In this study, two predictive models for myocardial ischemia were developed and evaluated using a training dataset and a validation dataset. The first model was established using the highest performing classification algorithm, based on all available clinical features. The second model, on the other hand, was established using the optimal classification algorithm based on a reduced set of clinical features obtained through the Recursive Feature Elimination (RFE) approach as previously described. Results showed that while the performance of the second model was slightly inferior compared to that of the first model, it presented a more parsimonious solution and thus, could be considered more practical for clinical applications.

Reference

1. Thoresen M. [Logistic regression - applied and applicable]. *Tidsskr Nor Laegeforen* 2017; **137**(19).

2. Rigatti SJ. Random Forest. *J Insur Med* 2017; **47**(1): 31-9.

3. Chen T, Guestrin C. XGBoost: A Scalable Tree Boosting System. *ACM* 2016.

4. Hastie T, Tibshirani R, Friedman J. The elements of statistical learning. Data mining, inference, and prediction. 2nd ed.

5. CristianintNello. An introduction to support vector machines and other kernel-based learning methods: An introduction to support vector machines and other kernel-based learning methods; 2000.

6. Lever J. Classification evaluation: It is important to understand both what a classification metric expresses and what it hides. *Nature methods* 2016; **13**(8): 603-5.

7. Greenwell BM, Boehmke BC, Mccarthy AJ. A Simple and Effective Model-Based Variable Importance Measure. 2018.

8. Saeys Y, Inza I, Larranaga P. A review of feature selection techniques in bioinformatics. *Bioinformatics* 2007; (19): 23.

9. Etay Z, Olga T, Donna MF, Jb A, Chris PH, Duan X. Recursive feature elimination. 2013.
