# Supplementary material for: Machine learning-based prediction model for myocardial ischemia under high altitude exposure: a cohort study
Source: Sci Rep. 2024 Jan 6;14:686. doi: 10.1038/s41598-024-51202-8 (PMC10770400; doi:10.1038/s41598-024-51202-8)
Supplement: Supplementary file 2 — Supplementary Tables. [file 41598_2024_51202_MOESM2_ESM.docx]

Table S1: Characteristics of participants in the training and test datasets

|  | **Training dataset** | | | | **Test dataset** | | | |
| --- | --- | --- | --- | --- | --- | --- | --- | --- |
|  | **No** | **Yes** | **OR** | **P** | **No** | **Yes** | **OR** | **P** |
|  | ***N=1964*** | ***N=177*** | **(95% CI)** |  | ***N=656*** | ***N=58*** | **(95% CI)** |  |
| Age(y) | 23⋅0 [22⋅0;26⋅0] | 24⋅0 [22⋅0;29⋅0] | 1⋅07 [1⋅04;1⋅10] | 0⋅001 | 24⋅0 [22⋅0;27⋅0] | 24⋅0 [22⋅0;27⋅8] | 1⋅03 [0⋅97;1⋅09] | 0⋅313 |
| Sex: |  |  |  | 0⋅005 |  |  |  | 1⋅000 |
| Female | 26 (1⋅32%) | 8 (4⋅52%) | 3⋅57 [1⋅48;7⋅72] | - | 11 (1⋅68%) | 0 (0⋅00%) | ⋅⋅ | - |
| Male | 1938 (98⋅7%) | 169 (95⋅5%) | Ref⋅ | - | 645 (98⋅3%) | 58 (100%) | Ref⋅ | - |
| Smoking duration |  |  |  | 0⋅012 |  |  |  | 0⋅274 |
| None | 1073 (54⋅6%) | 92 (52⋅0%) | Ref⋅ | - | 384 (58⋅5%) | 28 (48⋅3%) | Ref⋅ | - |
| <10 years | 779 (39⋅7%) | 65 (36⋅7%) | 0⋅97 [0⋅70;1⋅35] | - | 218 (33⋅2%) | 25 (43⋅1%) | 1⋅57 [0⋅89;2⋅77] | - |
| ≥10 years | 112 (5⋅70%) | 20 (11⋅3%) | 2⋅09 [1⋅21;3⋅46] | - | 54 (8⋅23%) | 5 (8⋅62%) | 1⋅30 [0⋅42;3⋅27] | - |
| Chesttightness.or.Chestpain |  |  |  | 0⋅002 |  |  |  | 0⋅268 |
| No | 1867 (95⋅1%) | 158 (89⋅3%) | Ref⋅ | - | 614 (93⋅6%) | 52 (89⋅7%) | Ref⋅ | - |
| Yes | 97 (4⋅94%) | 19 (10⋅7%) | 2⋅33 [1⋅35;3⋅83] | - | 42 (6⋅40%) | 6 (10⋅3%) | 1⋅72 [0⋅62;3⋅98] | - |
| BMI（kg/m^2^） | 22⋅1 [20⋅8;23⋅6] | 22⋅6 [21⋅0;24⋅4] | 1⋅09 [1⋅02;1⋅16] | 0⋅023 | 22⋅2 [20⋅8;23⋅8] | 22⋅5 [20⋅7;23⋅7] | 1⋅02 [0⋅90;1⋅15] | 0⋅680 |
| SBP（mmHg） | 116 [110;120] | 118 [110;124] | 1⋅00 [1⋅00;1⋅01] | 0⋅018 | 118 [110;121] | 120 [110;130] | 1⋅04 [1⋅02;1⋅07] | 0⋅006 |
| DBP（mmHg） | 80⋅0 [73⋅0;84⋅0] | 80⋅0 [75⋅0;85⋅0] | 1⋅01 [0⋅99;1⋅03] | 0⋅436 | 80⋅0 [73⋅0;84⋅0] | 81⋅5 [77⋅0;88⋅0] | 1⋅05 [1⋅02;1⋅08] | 0⋅002 |
| HR（min^−1^） | 80⋅0 [71⋅0;89⋅0] | 80⋅0 [75⋅0;88⋅0] | 1⋅00 [0⋅99;1⋅02] | 0⋅537 | 79⋅0 [70⋅0;88⋅0] | 78⋅5 [69⋅0;85⋅8] | 0⋅99 [0⋅97;1⋅01] | 0⋅486 |
| OS（%） | 93⋅0 [92⋅0;95⋅0] | 93⋅0 [92⋅0;96⋅0] | 1⋅01 [0⋅97;1⋅04] | 0⋅159 | 93⋅0 [92⋅0;95⋅0] | 93⋅0 [92⋅0;95⋅0] | 1⋅00 [0⋅99;1⋅01] | 0⋅132 |
| Highland acclimatization training*: |  |  |  | 0⋅986 |  |  |  | 1⋅000 |
| No | 151 (7⋅69%) | 13 (7⋅34%) | Ref⋅ | - | 37 (5⋅64%) | 3 (5⋅17%) | Ref⋅ | - |
| Yes | 1813 (92⋅3%) | 164 (92⋅7%) | 1⋅04 [0⋅60;1⋅97] | - | 619 (94⋅4%) | 55 (94⋅8%) | 1⋅05 [0⋅36;4⋅61] | - |
| Altitude of original station（m） |  |  |  | 0⋅002 |  |  |  | 0⋅202 |
| < 1500 | 1830 (93⋅2%) | 152 (85⋅9%) | Ref⋅ | - | 593 (90⋅4%) | 50 (86⋅2%) | Ref⋅ | - |
| 1500~2500 | 114 (5⋅80%) | 23 (13⋅0%) | 2⋅44 [1⋅48;3⋅87] | - | 51 (7⋅77%) | 8 (13⋅8%) | ⋅⋅ | - |
| >2500 | 20 (1⋅02%) | 2 (1⋅13%) | 1⋅29 [0⋅19;4⋅49] | - | 12 (1⋅83%) | 0 (0⋅00%) | ⋅⋅ | - |
| 3km test in Non-High Altitude (Score)^#^ | 77⋅0 [65⋅0;91⋅0] | 82⋅0 [62⋅0;93⋅0] | 1⋅00 [0⋅99;1⋅00] | 0⋅414 | 77⋅0 [65⋅0;91⋅0] | 81⋅0 [0⋅00;90⋅0] | 1⋅00 [0⋅99;1⋅00] | 0⋅944 |
| Sit-ups in Non-High Altitude (Score)^#^ | 93⋅0 [85⋅0;101] | 95⋅0 [88⋅0;101] | 1⋅01 [1⋅00;1⋅02] | 0⋅173 | 93⋅0 [85⋅0;101] | 93⋅0 [86⋅2;101] | 1⋅00 [0⋅98;1⋅02] | 0⋅814 |
| Serpentine Run in Non-High Altitude (Score)^#^ | 90⋅0 [75⋅0;96⋅0] | 90⋅0 [78⋅0;96⋅0] | 1⋅00 [0⋅99;1⋅01] | 0⋅923 | 90⋅0 [75⋅0;96⋅0] | 83⋅0 [72⋅5;95⋅0] | 0⋅99 [0⋅98;1⋅00] | 0⋅195 |
| SCOPA-AUT SCORE in Non-High Altitude | 4⋅00 [2⋅00;8⋅00] | 3⋅00 [2⋅00;7⋅00] | 0⋅98 [0⋅95;1⋅02] | 0⋅170 | 4⋅00 [2⋅00;8⋅00] | 5⋅00 [3⋅00;8⋅00] | 1⋅03 [0⋅97;1⋅10] | 0⋅121 |
| ALB(g/L) | 47⋅4 [45⋅1;48⋅8] | 46⋅5 [45⋅1;48⋅1] | 0⋅92 [0⋅87;0⋅98] | 0⋅012 | 47⋅4 [45⋅1;48⋅8] | 46⋅5 [45⋅2;48⋅9] | 0⋅92 [0⋅84;1⋅02] | 0⋅212 |
| ALP(U/L) | 59⋅0 [51⋅0;73⋅0] | 62⋅0 [50⋅0;67⋅0] | 0⋅98 [0⋅97;0⋅99] | 0⋅020 | 59⋅0 [51⋅0;73⋅0] | 62⋅0 [50⋅0;69⋅8] | 1⋅00 [0⋅98;1⋅02] | 0⋅323 |
| ALT(U/L) | 18⋅0 [15⋅0;29⋅0] | 19⋅0 [15⋅0;30⋅0] | 0⋅99 [0⋅99;1⋅00] | 0⋅532 | 19⋅0 [15⋅0;27⋅0] | 18⋅0 [15⋅0;29⋅8] | 0⋅99 [0⋅98;1⋅00] | 0⋅816 |
| TBA(μmol/l) | 3⋅80 [3⋅10;6⋅00] | 3⋅50 [2⋅00;7⋅60] | 1⋅01 [0⋅98;1⋅04] | 0⋅003 | 3⋅60 [2⋅90;6⋅00] | 3⋅30 [1⋅90;4⋅02] | 0⋅96 [0⋅91;1⋅03] | <0⋅001 |
| Baso.Per(%) | 0⋅40 [0⋅30;0⋅60] | 0⋅50 [0⋅30;0⋅60] | 2⋅64 [1⋅44;4⋅84] | 0⋅002 | 0⋅40 [0⋅30;0⋅60] | 0⋅40 [0⋅30;0⋅58] | 2⋅03 [0⋅66;6⋅24] | 0⋅412 |
| Ca(mmol/L) | 2⋅37 [2⋅31;2⋅41] | 2⋅42 [2⋅32;2⋅47] | 2⋅23  [1⋅88;3⋅63] | <0⋅001 | 2⋅37 [2⋅30;2⋅41] | 2⋅38 [2⋅31;2⋅47] | 1⋅75 [1⋅04;2⋅32] | 0⋅036 |
| CHE(U/L) | 8704 [7420;9258] | 8685 [7550;10585] | 1⋅00 [1⋅00;1⋅00] | 0⋅145 | 8705 [7410;9453] | 8812 [7222;10466] | 1⋅00 [1⋅00;1⋅00] | 0⋅959 |
| CO_2_CP(mmo/L) | 27⋅2 [25⋅4;28⋅2] | 27⋅6 [26⋅4;28⋅5] | 1⋅10 [1⋅02;1⋅19] | 0⋅024 | 27⋅1 [25⋅1;28⋅2] | 27⋅5 [26⋅3;28⋅3] | 1⋅15 [1⋅01;1⋅31] | 0⋅123 |
| CysC(mg/L) | 0⋅89 [0⋅82;0⋅92] | 0⋅90 [0⋅81;0⋅95] | 0⋅35 [0⋅06;1⋅97] | 0⋅673 | 0⋅88 [0⋅82;0⋅92] | 0⋅92 [0⋅81;0⋅96] | 0⋅79 [0⋅04;16⋅4] | 0⋅637 |
| Crea(μmol/L) | 88⋅0 [80⋅0;97⋅0] | 83⋅0 [79⋅0;93⋅0] | 0⋅97 [0⋅95;0⋅98] | <0⋅001 | 89⋅0 [81⋅0;97⋅0] | 85⋅5 [79⋅0;95⋅0] | 0⋅98 [0⋅95;1⋅01] | 0⋅100 |
| DBIL(μmol/L) | 4⋅20 [3⋅60;6⋅20] | 4⋅90 [3⋅90;6⋅00] | 1⋅03 [0⋅96;1⋅09] | 0⋅028 | 4⋅20 [3⋅60;6⋅30] | 5⋅60 [4⋅40;6⋅97] | 1⋅18 [1⋅07;1⋅31] | <0⋅001 |
| Eo(*10^9^/L) | 0⋅15 [0⋅09;0⋅19] | 0⋅16 [0⋅08;0⋅37] | 40⋅5 [15⋅1;109] | 0⋅015 | 0⋅15 [0⋅09;0⋅19] | 0⋅15 [0⋅05;0⋅36] | 20⋅4 [3⋅98;104] | 0⋅837 |
| Eos.Per(%) | 2⋅20 [1⋅60;3⋅00] | 2⋅60 [1⋅30;5⋅00] | 1⋅28 [1⋅20;1⋅36] | <0⋅001 | 2⋅20 [1⋅50;3⋅00] | 2⋅00 [1⋅10;4⋅92] | 1⋅23 [1⋅10;1⋅36] | 0⋅608 |
| G(g/L) | 25⋅6 [23⋅6;27⋅3] | 27⋅8 [25⋅6;28⋅5] | 1⋅22 [1⋅16;1⋅29] | <0⋅001 | 25⋅6 [22⋅9;27⋅3] | 27⋅8 [25⋅9;28⋅5] | 1⋅20 [1⋅11;1⋅30] | <0⋅001 |
| GGT(U/L) | 16⋅0 [13⋅0;23⋅0] | 18⋅0 [14⋅0;30⋅0] | 1⋅00 [0⋅99;1⋅01] | 0⋅057 | 16⋅0 [13⋅0;23⋅0] | 17⋅0 [14⋅0;28⋅0] | 0⋅99 [0⋅98;1⋅01] | 0⋅404 |
| GLU (mmol/L) | 4⋅03 [3⋅53;4⋅47] | 4⋅33 [4⋅10;4⋅59] | 2⋅11 [1⋅73;2⋅58] | <0⋅001 | 4⋅03 [3⋅53;4⋅47] | 4⋅33 [3⋅99;4⋅59] | 1⋅92 [1⋅37;2⋅67] | <0⋅001 |
| HCT(%) | 45⋅2 [43⋅5;47⋅9] | 45⋅9 [44⋅5;46⋅9] | 1⋅04 [0⋅99;1⋅08] | 0⋅060 | 45⋅5 [43⋅9;47⋅6] | 44⋅9 [44⋅2;46⋅9] | 0⋅98 [0⋅91;1⋅06] | 0⋅681 |
| HGB(g/L) | 153 [146;162] | 150 [148;160] | 1⋅00 [0⋅99;1⋅01] | 0⋅967 | 154 [146;162] | 149 [147;156] | 0⋅99 [0⋅97;1⋅01] | 0⋅222 |
| Lymph.Per(%) | 35⋅9 (5⋅48) | 34⋅7 (6⋅04) | 0⋅96 [0⋅94;0⋅99] | 0⋅011 | 36⋅0 (5⋅55) | 35⋅0 (6⋅30) | 0⋅97 [0⋅92;1⋅01] | 0⋅221 |
| MCHC(g/L) | 338 (9⋅00) | 332 (24⋅5) | 0⋅95 [0⋅94;0⋅97] | 0⋅001 | 338 (9⋅25) | 335 (10⋅1) | 0⋅97 [0⋅94;1⋅00] | 0⋅034 |
| MCV (fL) | 88⋅9 [87⋅5;91⋅5] | 90⋅6 [86⋅5;92⋅0] | 1⋅01 [0⋅98;1⋅05] | 0⋅617 | 88⋅9 [87⋅5;91⋅4] | 89⋅2 [86⋅5;92⋅0] | 0⋅99 [0⋅94;1⋅04] | 0⋅922 |
| Mono.Per(%) | 6⋅70 [5⋅40;7⋅70] | 6⋅60 [5⋅50;7⋅40] | 0⋅93 [0⋅84;1⋅03] | 0⋅141 | 6⋅70 [5⋅40;7⋅70] | 7⋅10 [5⋅55;7⋅80] | 1⋅07 [0⋅92;1⋅24] | 0⋅467 |
| Na(mmol/L) | 144 [143;146] | 144 [144;145] | 0⋅96 [0⋅89;1⋅05] | 0⋅563 | 144 [143;146] | 144 [143;144] | 0⋅86 [0⋅75;0⋅99] | 0⋅067 |
| Neu.Per(%) | 55⋅3 [49⋅8;57⋅8] | 54⋅1 [50⋅5;57⋅2] | 1⋅00 [0⋅98;1⋅03] | 0⋅732 | 55⋅3 [49⋅8;57⋅8] | 53⋅0 [50⋅1;58⋅2] | 0⋅99 [0⋅95;1⋅04] | 0⋅428 |
| P(mmol/L) | 1⋅19 [1⋅08;1⋅27] | 1⋅09 [1⋅05;1⋅14] | 0⋅11 [0⋅04;0⋅31] | <0⋅001 | 1⋅17 [1⋅07;1⋅25] | 1⋅10 [1⋅06;1⋅14] | 0⋅08 [0⋅01;0⋅47] | 0⋅001 |
| PA (mg/L) | 312 [289;334] | 312 [296;341] | 1⋅00 [1⋅00;1⋅01] | 0⋅233 | 316 [294;334] | 316 [290;340] | 1⋅00 [0⋅99;1⋅01] | 0⋅592 |
| PCT (%) | 0⋅22 [0⋅20;0⋅25] | 0⋅22 [0⋅19;0⋅24] | 0⋅00 [0⋅00;0⋅08] | 0⋅021 | 0⋅22 [0⋅20;0⋅25] | 0⋅21 [0⋅18;0⋅24] | 0⋅00 [0⋅00;0⋅31] | 0⋅034 |
| PDW(fL) | 14⋅4 (2⋅29) | 14⋅7 (2⋅11) | 1⋅07 [1⋅00;1⋅15] | 0⋅039 | 14⋅4 (2⋅28) | 14⋅5 (2⋅35) | 1⋅02 [0⋅91;1⋅15] | 0⋅700 |
| PLT(*10^9^/L) | 230 (37⋅5) | 214 (45⋅3) | 0⋅99 [0⋅99;0⋅99] | <0⋅001 | 230 (37⋅7) | 211 (49⋅6) | 0⋅99 [0⋅98;0⋅99] | 0⋅005 |
| RBC(*10^12^/L) | 5⋅08 [4⋅86;5⋅33] | 5⋅10 [4⋅89;5⋅39] | 1⋅31 [0⋅91;1⋅89] | 0⋅066 | 5⋅14 [4⋅88;5⋅33] | 5⋅08 [4⋅83;5⋅20] | 1⋅04 [0⋅51;2⋅13] | 0⋅557 |
| RBP(mg/L) | 40⋅2 [35⋅6;42⋅3] | 41⋅3 [35⋅3;43⋅1] | 1⋅00 [0⋅97;1⋅03] | 0⋅356 | 41⋅2 [36⋅3;43⋅0] | 41⋅5 [35⋅7;44⋅4] | 1⋅02 [0⋅97;1⋅07] | 0⋅232 |
| RDW.CV (%) | 13⋅1 [12⋅7;13⋅6] | 12⋅9 [12⋅7;13⋅4] | 0⋅97 [0⋅83;1⋅14] | 0⋅003 | 13⋅1 [12⋅7;13⋅6] | 12⋅7 [12⋅5;13⋅2] | 0⋅67 [0⋅45;1⋅01] | <0⋅001 |
| RDW.SD (fL) | 41⋅1 [40⋅3;44⋅2] | 41⋅6 [40⋅2;42⋅4] | 0⋅94 [0⋅88;1⋅00] | 0⋅420 | 41⋅1 [40⋅2;44⋅1] | 41⋅4 [39⋅9;41⋅7] | 0⋅90 [0⋅80;1⋅00] | 0⋅124 |
| AST(U/L) | 22⋅0 [19⋅0;30⋅0] | 20⋅0 [18⋅0;26⋅0] | 0⋅98 [0⋅96;0⋅99] | <0⋅001 | 22⋅0 [19⋅0;29⋅0] | 19⋅0 [17⋅0;24⋅0] | 0⋅95 [0⋅92;0⋅99] | <0⋅001 |
| TP(g/L) | 72⋅0 [70⋅7;74⋅6] | 73⋅7 [70⋅7;75⋅5] | 1⋅11 [1⋅06;1⋅16] | <0⋅001 | 71⋅5 [69⋅9;74⋅6] | 74⋅1 [70⋅9;75⋅6] | 1⋅11 [1⋅03;1⋅19] | 0⋅014 |
| UA(μmol/L) | 383 [320;427] | 377 [326;417] | 1⋅00 [1⋅00;1⋅00] | 0⋅615 | 380 [320;427] | 377 [341;396] | 1⋅00 [1⋅00;1⋅00] | 0⋅785 |
| Urea(mmol/L) | 5⋅10 [4⋅40;5⋅70] | 5⋅50 [5⋅00;6⋅30] | 1⋅35 [1⋅18;1⋅53] | <0⋅001 | 5⋅10 [4⋅60;5⋅60] | 5⋅90 [5⋅03;7⋅22] | 1⋅65 [1⋅32;2⋅06] | <0⋅001 |
| WBC(*10^9^/L) | 6⋅75 [5⋅84;7⋅36] | 5⋅91 [5⋅29;7⋅26] | 0⋅74 [0⋅65;0⋅85] | <0⋅001 | 6⋅59 [5⋅70;7⋅36] | 5⋅90 [5⋅51;6⋅86] | 0⋅90 [0⋅72;1⋅13] | 0⋅022 |

Continuous data conforming to a normal distribution were reported as mean (SD) and those not conforming as median(quartiles). Odds ratios and 95% CIs were calculated, either using count data for categorical variables or a logistic regression model for continuous variables. Statistical significance was determined using t-test, Rank-sum test or χ² test, with p-values calculated.

Non-High Altitude: an altitude <2500m. High Altitude: an altitude≥2500m. BMI: body mass index. SBP: systolic blood pressure. DBP: diastolic blood pressure. HR: heart rate. OS: oxygen saturation. SCOPA-AUT: the Scale for Outcomes in Parkinson′s Disease for Autonomic Symptoms. ALB:albumin. ALP: alkaline phosphatase. ALT: alanine transaminase. Baso.Per: basophile granulocyte percentage. Ca: calcium. CHE: Cholinesterase. CO_2_CP: carbon dioxide binding capacity. CysC: serum cystatin C. DBIL: direct bilirubin. Eo: eosinophils. Eos.Per: eosinophils percentage. G: globulin. GGT: γ-glutamyl transpeptidase. GLU: glucose. HCT:Hematocrit. HGB: hemoglobin. Lymph.per: lymphocytes percentage. MCHC: mean corpuscular hemoglobin concentration. MCV:Mean Corpuscular Volume. Mono.Per: monocytes percentage. Neu.Per: neutrophils percentage. P: phosphorus. PA: Prealbumin. PCT: thrombocytocrit. PDW: platelet distributing width. PLT: platelet. RBC:red blood cell. RBP:retinol conjugated protein. RDW.CV: Coefficient of variation of red blood cell distribution width. RDW.SD: Standard deviation of red blood cell distribution width. TBA: total bile acid. TP: total protein. UA: Uric acid. WBC: white blood cell.

^*^Participants entered the destination after 1 month of highland acclimatization training at an altitude of 3000m.

^#^ The calculation of physical ability scores was based on Military Common Subject Training Program.

Table S2: Metrics of each algorithm in the training dataset and test dataset.

|  | Training datasets | | |  | | Test datasets | | |  |
| --- | --- | --- | --- | --- | --- | --- | --- | --- | --- |
| Model | Mean AUC | Mean Accuracy | Mean Precision | | AUC | | Accuracy | Precision | |
| Logistic Regression | 0⋅81 | 0⋅81 | 0⋅56 | | 0⋅79 | | 0⋅92 | 0⋅59 | |
| Random Forest | 0⋅86 | 0⋅86 | 1 | | 0⋅86 | | 0⋅95 | 1 | |
| XGBoost | 0⋅86 | 0⋅86 | 0⋅9 | | 0⋅85 | | 0⋅95 | 0⋅95 | |
| Knn | 0⋅75 | 0⋅75 | 0⋅44 | | 0⋅76 | | 0⋅89 | 0⋅34 | |
| SVM | 0⋅76 | 0⋅76 | 0 | | 0⋅75 | | 0⋅92 | 0 | |

Table S3. Importance of variables for each algorithm.

| Variable | Importance | Algorithm |
| --- | --- | --- |
| Eos.Per | 28⋅43134715 | RF |
| G | 22⋅80477863 | RF |
| Ca | 19⋅45259241 | RF |
| PLT | 18⋅52196498 | RF |
| GLU | 18⋅35056829 | RF |
| AST | 15⋅70426751 | RF |
| P | 14⋅83644605 | RF |
| TP | 14⋅51145382 | RF |
| MCHC | 13⋅84252216 | RF |
| Urea | 12⋅98973509 | RF |
| Crea | 12⋅90816393 | RF |
| WBC | 12⋅86365054 | RF |
| SBP | 6⋅906242203 | RF |
| BMI | 6⋅518002665 | RF |
| Age | 6⋅489505943 | RF |
| HR | 5⋅88633716 | RF |
| Serpentine Run in Non-High Altitude (Score) | 5⋅832592154 | RF |
| 3km test in Non-High Altitude (Score) | 5⋅18842319 | RF |
| Sit-ups in Non-High Altitude (Score) | 5⋅05842638 | RF |
| SCOPA-AUT SCORE in Non-High Altitude | 4⋅905252061 | RF |
| OS | 4⋅773505085 | RF |
| DBP | 4⋅415754099 | RF |
| Altitude of original station_1500~2500m | 3⋅568954898 | RF |
| Smoking duration_≥10 years | 1⋅606936477 | RF |
| Chesttightness or Chestpain_Yes | 0⋅995133879 | RF |
| Sex_Male | 0⋅741734586 | RF |
| Smoking duration_None | 0⋅563864626 | RF |
| Altitude of original station_X≥2500m | 0⋅348531245 | RF |
| Highland acclimatization training_Yes | 0⋅227611573 | RF |
| Eos.Per | 0⋅108874962 | XGBoost |
| Ca | 0⋅104427389 | XGBoost |
| GLU | 0⋅103004845 | XGBoost |
| P | 0⋅09087377 | XGBoost |
| G | 0⋅077995543 | XGBoost |
| PLT | 0⋅0651852 | XGBoost |
| MCHC | 0⋅054797765 | XGBoost |
| AST | 0⋅048047165 | XGBoost |
| Crea | 0⋅045011601 | XGBoost |
| 3km test in Non-High Altitude (Score) | 0⋅041803013 | XGBoost |
| TP | 0⋅037397062 | XGBoost |
| WBC | 0⋅028245819 | XGBoost |
| SBP | 0⋅027184186 | XGBoost |
| Altitude of original station_1500⋅2500m | 0⋅02683664 | XGBoost |
| Urea | 0⋅024074554 | XGBoost |
| Age | 0⋅020510716 | XGBoost |
| BMI | 0⋅018361114 | XGBoost |
| HR | 0⋅01253739 | XGBoost |
| SCOPA-AUT SCORE in Non-High Altitude | 0⋅012398312 | XGBoost |
| Sit-ups in Non-High Altitude (Score) | 0⋅011079955 | XGBoost |
| Serpentine Run in Non-High Altitude (Score) | 0⋅009831648 | XGBoost |
| Smoking duration_≥10 years | 0⋅009657617 | XGBoost |
| DBP | 0⋅008471135 | XGBoost |
| OS | 0⋅008080452 | XGBoost |
| Smoking duration_None | 0⋅004088921 | XGBoost |
| Highland acclimatization training_Yes | 0⋅001223224 | XGBoost |
| G | 6⋅419666407 | LR |
| Eos⋅Per | 5⋅592433824 | LR |
| GLU | 5⋅404094608 | LR |
| Ca | 3⋅632597082 | LR |
| MCHC | 3⋅588344201 | LR |
| PLT | 3⋅543722391 | LR |
| Altitude of original station_1500~2500m | 2⋅977694791 | LR |
| Crea | 2⋅808583353 | LR |
| TP | 2⋅770319387 | LR |
| Age | 2⋅634129573 | LR |
| AST | 2⋅500049255 | LR |
| Smoking duration_≥10 years | 1⋅668636665 | LR |
| Chesttightness or Chestpain_Yes | 1⋅454848637 | LR |
| SCOPA-AUT SCORE in Non-High Altitude | 1⋅374717084 | LR |
| WBC | 1⋅23905244 | LR |
| SBP | 1⋅065359601 | LR |
| BMI | 0⋅990694343 | LR |
| 3km test in Non-High Altitude (Score) | 0⋅62843196 | LR |
| P | 0⋅52352223 | LR |
| Sit-ups in Non-High Altitude (Score) | 0⋅419152318 | LR |
| Serpentine Run in Non-High Altitude (Score) | 0⋅40385764 | LR |
| DBP | 0⋅242195737 | LR |
| Sex_Male | 0⋅235646863 | LR |
| Smoking duration_None | 0⋅219428107 | LR |
| Urea | 0⋅212293322 | LR |
| Altitude of original station_≥2500m | 0⋅159935849 | LR |
| Highland acclimatization training_Yes | 0⋅044987913 | LR |
| OS | 0⋅040760722 | LR |
| HR | 0⋅033009502 | LR |

RF:random forest. LR:logistic regression.

Table S4. Slope of calibration curve in RF.5F and RF.27F models.

| Upper | Mean | Lower | Model | Dataset |
| --- | --- | --- | --- | --- |
| 2⋅241566034 | 1⋅311495626 | 0⋅381425218 | RF.5F | Training dataset |
| 1⋅124157414 | 0⋅903848438 | 0⋅683539463 | RF.27F | Training dataset |
| 1⋅536413484 | 1⋅170884695 | 0⋅805355906 | RF.5F | Test data |
| 1⋅052316295 | 0⋅837448423 | 0⋅62258055 | RF.27F | Test data |

Table S5. Intercept of calibration curve in RF.5F and RF.27F models.

| Upper | Mean | Lower | Model | Dataset |
| --- | --- | --- | --- | --- |
| 0⋅032690801 | 0⋅021798498 | 0⋅010906194 | RF.5F | Training dataset |
| 0⋅068172008 | 0⋅053323841 | 0⋅038475675 | RF.27F | Training dataset |
| 0⋅060456264 | 0⋅042677907 | 0⋅02489955 | RF.5F | Test data |
| 0⋅053630777 | 0⋅040927524 | 0⋅028224271 | RF.27F | Test data |
