# Supplementary material for: Machine learning-based prediction model for myocardial ischemia under high altitude exposure: a cohort study
Source: Sci Rep. 2024 Jan 6;14:686. doi: 10.1038/s41598-024-51202-8 (PMC10770400; doi:10.1038/s41598-024-51202-8)
Supplement: Supplementary file 3 — Supplementary Figure S1. [file 41598_2024_51202_MOESM3_ESM.docx]

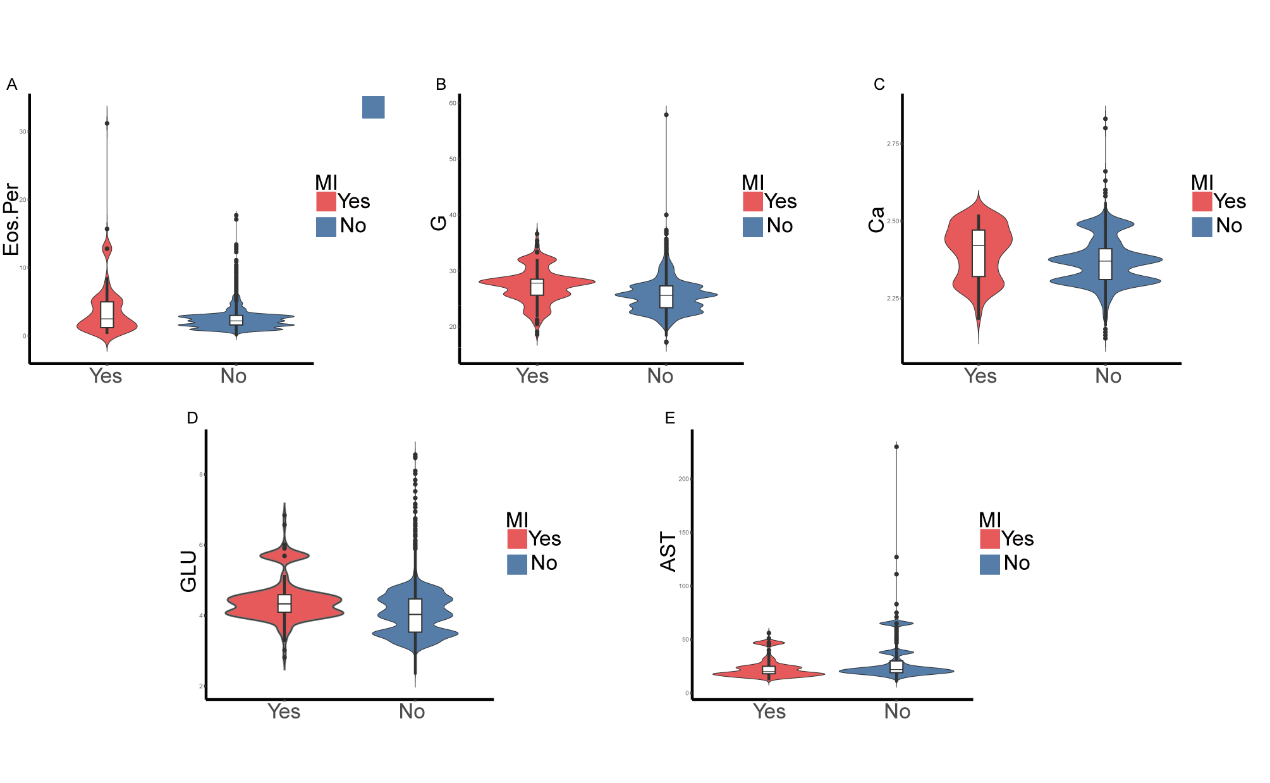


**Figure S1:Top 5 predictive features selected under RFE algorithm.**

The top 5 predictive features selected by the RFE algorithm are Eos.Per, G, Ca, GLU, and TBA, represented in the form of violin plots, showing the data distribution and its probability density of the entire dataset. The middle box represents the range of quartiles, and the thin black lines extending from it represent the data range, with the ends being the maximum and minimum values, while the black line in the middle of the box represents the median. The width of the violin plot represents the probability of that value appearing in the total population shown. RFE:Recursive Feature Elimination. Ca: calcium. Eos.Per: eosinophils percentage. G: globulin. GLU: glucose. AST: Aspartate aminotransferase.
